# A comparison of attitudes towards animal welfare between British and Japanese zoo visitors: Where and when do cultural differences diverge?

**DOI:** 10.1371/journal.pone.0320241

**Published:** 2025-04-08

**Authors:** Yumi Yamanashi, Yuko Ikkatai, Moe Honjo, Nahoko Tokuyama, Rie Akami, Duncan Andrew Wilson, Hannah M. Buchanan-Smith

**Affiliations:** 1 Center for Research and Education of Wildlife, Kyoto City Zoo, Kyoto, Japan; 2 Wildlife Research Center, Kyoto University, Kyoto, Japan; 3 Research Institute for Humanity and Nature, Kyoto, Japan; 4 Institute of Human and Social Sciences, Kanazawa University, Kanazawa, Japan; 5 Graduate School of Fisheries and Environmental Sciences, Nagasaki University, Nagasaki, Japan; 6 Japan Monkey Centre, Aichi, Japan; 7 Department of Psychology, Graduate School of Letters, Kyoto University, Kyoto, Japan; 8 Faculty of Natural Sciences, University of Stirling, Stirling, Scotland, United Kingdom; University Centre Sparsholt, UNITED KINGDOM OF GREAT BRITAIN AND NORTHERN IRELAND

## Abstract

Scientific evidence should form the basis for policy and practice decisions concerning animal welfare. However, cultural attitudes inevitably influence decision-making processes. We conducted a survey of general attitudes towards the welfare of zoo-housed animals, live prey feeding and trust in zoo management in British and Japanese zoo visitors (1,611 visitors aged over six years from one British zoo and two Japanese zoos). We asked respondents about their general attitudes towards animals, concepts of animal welfare, and acceptance of using a range of vertebrates and invertebrates as live prey. Overall, both British and Japanese respondents were concerned about animal welfare. However, when considering what is important for animal welfare, Japanese respondents mostly limited responses to basic factors such as food and sociality, whilst British respondents referred more to providing stimulation in the captive environment and positive emotions. The level of tolerance regarding live prey feeding was similar between countries, except for feeding octopuses which was less acceptable to British zoo visitors. Respondents differed in their reasons for this distinction; Japanese respondents often referred to personal preference and feelings in deciding which prey is acceptable to live feed, while British respondents often referred to animal intelligence and behavioural and life complexities. The tendency in trust in governing countries, zoos, and caregivers was similar between the two countries. Overall, British and Japanese respondents showed many similar views, but Japanese respondents tended to make more subjective decisions than British respondents. These attitudes are not directly reflected in legislation concerning animal welfare in each country.

## Introduction

Moral systems are universal among humans, and it has been argued that they can also apply to other social animals living in long-lasting social groups [1]. Human moral attitudes also extend to nonhuman animals (hereafter animals); however, the realization of compassion for animals in society differs across countries and regions. The concept of animal welfare has been adopted as a professional norm in zoo and aquarium facilities worldwide [2]. The definition of animal welfare is commonly expressed as ‘*the physical and mental state of an animal in relation to the conditions in which it lives and dies*’ [3, p. 1] and can be scientifically evaluated. Policy and practice decisions regarding animal welfare should be based on scientific evidence. However, the decision-making and implementation process can inevitably be influenced by various factors such as ethical attitudes, religion, social structures, education, age, gender, past experiences with animals and the surrounding environment [4–6]. In contrast to Western countries where the concept of animal welfare was mainly developed and used as the basis for laws [1,7,8], in Japan, a different term, ‘Animal Aigo 動物愛護,’ meaning ‘loving, not killing and protecting animals’, is widely used for laws protecting animals [9]. Animal aigo emerged in the early 20th century as a response to the anti-cruelty movement introduced by Westerners, a movement deeply rooted in early 19th-century British thought [10]. The concept of animal welfare arrived later in Japan [11], and researchers have sometimes observed confusion between Aigo and welfare among the general public and even among specialists [12]. Aigo is predominantly rooted in human subjectivity, and similar attitudes may be prevalent in other countries, as public reactions to moral issues often derive primarily from moral intuitions rather than moral reasoning [13], even though they may not be explicitly defined. Animal welfare scientists have also attempted to address public concerns about welfare, but conflicts can arise, as public perceptions may not always align with scientific evidence. Therefore, understanding how animals are perceived in each society is important for facilitating discussion and implementing strategies to improve animal welfare.

Some studies have directly investigated attitudes toward animal welfare across countries [14–16]. However, most studies have focused on farm or companion animal welfare [e.g., 14], with less attention given to wild animals under human care. Some studies have compared the attitudes of zoo and aquarium staff toward animal welfare or ethical issues across different countries [4,17] or occupations [18]. Other research has examined visitors’ perceptions of animals in specific zoos [19,20]. Notably, no study has been conducted to directly compare zoo visitors’ attitudes toward zoo animal welfare between different countries. Furthermore, in most studies, only adult respondents were included. Little is known about the emergence of cultural differences in attitudes toward animals across various countries. Differences in understanding and perception of animals between adults and children have sometimes been reported [21]. Children’s attitudes toward animals change throughout their development [22,23]. Kellert (1985) suggested that a significant shift in affective relationships with animals occurs from ages six to nine, and ethical concern for animals broadens as children reach ages 13 to 16 [23]. Neldner and Wilks (2022) synthesize studies that examine children’s perceptions of the moral worth of animals [22]. They found that children assign animals a high moral standing early in childhood, which decreases during late childhood, continues to diminish throughout adolescence, and into adulthood. Zoos offer a unique setting for investigating these developmental changes, as they are popular educational destinations for children to learn about animals.

Zoos are places where animals receive daily care, and visitors’ perspectives on the specific, practical caregiving decisions made offer valuable insights into moral attitudes toward animals. A common practice in many zoos is the feeding of live prey to animals to simulate their natural hunting behaviours in the wild. Live prey feeding is controversial, as the benefits and costs to animal welfare vary depending on whether one empathizes with the prey or the predator. Furthermore, species differences also influence how people make ethical decisions [14,24]. Ethically, it is sometimes recommended that live prey feeding be replaced with other forms of environmental enrichment. For instance, Quirke et al. [25] documented the speed of cheetahs using an enrichment activity called the ‘cheetah run’, which enables complex hunting behaviours similar to those observed in the wild without the use of live animals. However, live prey feeding may be deemed necessary in certain situations, such as to reintroduce animals back to the wild, or when animals refuse to eat unless the prey is alive. Due to this complexity, legislation regarding live prey feeding varies across countries [17]. In the UK, the question of whether animals possess sentience is employed as a criterion for the legal protection of species [26]. As of 2012, current legislation in the UK discourages live prey feeding of vertebrates [27], and there is ongoing discussion about prohibiting the live feeding of vertebrate prey in zoos [28]. In contrast, Japan does not have a comparable legal movement. The Japanese Ministry of the Environment requires zoos to reduce suffering and pain of prey animals in the case of live prey feeding [9]. The Japanese Association of Zoos and Aquariums (JAZA) has recently included the policy for live prey feeding in its animal welfare standards. However, live prey feeding is still considered as a necessary measure. The moral acceptability of live prey feeding has been investigated in some literature [17,29–31]. For example, Ings et al. (1997) examined zoo visitors’ perceptions of live prey feeding at Edinburgh Zoo and found that 96% of respondents agreed to the use of live insects for on-exhibit lizards. However, only 32% agreed to the use of live rabbits for on-exhibit cheetahs, with a higher proportion if it happened off-exhibit. Marshall et al. (2019) compared the attitudes of zoo and aquarium professionals toward live prey feeding of aquatic animals in the UK and the US, finding differences between countries [17]. However, the responses were not necessarily consistent with the views expressed in the laws of each country. Live prey feeding is a fascinating subject for exploring cultural perspectives on species-based ethical judgments related to animal welfare and how these perceptions influence legislation. However, there is currently no study on this topic in Japan, and there is a need for up-to-date information about UK zoos.

A crucial factor in whether the public can trust a zoo or aquarium is the ability of its management to provide for the welfare of its animals. A widely accepted definition of trust is ‘a psychological state comprising the intention to accept vulnerability based on positive expectations of the intentions or behaviours of another’ [32]. Rank et al. [33] reported that there were gaps in public perception and expectations regarding ethical integrity in zoos and aquariums. A failure to address animal welfare concerns can lead to a decrease in trust towards these institutions [34]. Simultaneously, animal welfare is a continuum rather than a discrete concept, and methods to enhance animal welfare can vary across situations. Consequently, there is always ambiguity in terms of what is best under given situations and consensus is not always reached. For instance, in the case of farm animals, farmers and the public may differ in their perceptions of animal welfare [35]. In a society with such complexity, researching and communicating about animal welfare necessitates trust. Therefore, trust and animal welfare can be considered an inextricable and bidirectional relationship. However, it is not clear whether this relationship is well understood and applicable to every culture.

The purpose of this study is to understand the similarities and differences in attitudes toward the welfare of zoo-housed animals between British and Japanese people, aiming to facilitate mutual understanding and promote constructive discussion within and across cultures. We selected Britain and Japan for this comparative analysis because of their distinct cultures and legislations. Britain, being where animal protection legislation was originally developed and the conceptualization of animal welfare advanced [1], contrasts with Japan, which has long adopted different approaches and terms. The Animal Protection Index (API) ranks 50 countries around the world according to their animal welfare policy and legislation and finds substantially better policy in the UK than Japan [36]. However, policies and legislation do not fully represent attitudes toward animals in each country. Specifically, our study focused on three key aspects. First, we compared general interest in and perception of zoo animal welfare and identified the factors that constitute animal welfare in each country. We hypothesized that both British and Japanese people would consider animal welfare to be important as it is a common human attribute to sympathize with non-human animals. However, we hypothesized that the specifics of what constitutes good animal welfare would differ between the countries, with Japanese opinions being more rooted in subjective perception, influenced by the culture of ‘Animal Aigo.’ We explored the development of such attitudes by age. Second, we asked about the acceptability of live prey feedings in various species and the reasons behind such decisions. We hypothesized that British and Japanese people would show differences across species and their underlying reasons. British people’s perception would align with the UK laws, while Japanese people’s perception would be more intuitive as there is no legislation specific to live prey feeding. Again, we investigated the development of attitudes. Third, we investigated how visitors judge and trust zoos and the governing country from the perspective of animal care. We hypothesized that British people would trust zoos and their governing country more than Japanese people as there are stricter laws and guidelines in the UK.

## Materials and methods

The onsite questionnaire surveys were conducted at a British zoo (Royal Zoological Society of Scotland (RZSS) Edinburgh Zoo) and two Japanese zoos (Kyoto City Zoo and Japan Monkey Centre). We collected a total of 1,611 responses (British zoo: N =  616, two Japanese zoos: N =  995 [Kyoto City Zoo: N = 792; Japan Monkey Centre: N = 203]). We collected data between 22 June and 3 July 2022 (7 days in total) at the British zoo, and from 24 October to 13 November 2021 and from 5 to 22 November 2022 (10 days in total) at the Japanese zoos. We utilized responses exclusively from British citizens at the British zoo. To collect responses only from British individuals at RZSS Edinburgh Zoo, we asked for their nationalities in advance and included a nationality question in the questionnaire to ensure accuracy. In addition to the 616 British respondents, 57 people from other nationalities or who did not provide nationality information were excluded from the analyses. In Japanese zoos, although we did not explicitly ask for nationalities, the questionnaire was administered in Japanese, suggesting that respondents were most likely Japanese. Respondents’ consent was obtained before conducting the survey, following the ethical guidelines of each organization and country. In Japan, consent was obtained verbally, and the questionnaire form was provided only if the participants agreed. In Britain, consent from adults and assent from children were obtained using forms with the participants’ signatures if they agreed. Questions were structured so that respondents did not have to answer if they did not wish to. All respondents were offered a postcard as a reward.

The questionnaire consisted of both open and closed questions, with specific questions shown in Tables 1 and S1 in S1 File which includes Japanese versions. Different questionnaires were administered to adults and two age groups of children (16 years and older, and 6 - 15 years old), modified to accommodate their age-appropriate level of understanding. The questionnaire asked respondents about their general attitudes towards zoo animal welfare (on a 4-point Likert scale and in free responses) and levels of tolerance towards using animals as live prey (i.e., rabbits, mice, chickens, frogs, goldfish, sardines, octopuses, crayfish, clams, crickets, and earthworms). We selected species from diverse taxa that could potentially be used as live prey. For adults, we also inquired whether they think caregivers, the zoo, or the governing country are fulfilling their responsibility in terms of animal welfare as a proxy to examine their trust. We limited the number of questions and used simplified texts, and images for children to facilitate understanding and survey completion. The adult questionnaire was two pages long with double-sided printing, while the children’s questionnaire was one page long.

**Table 1:** Questionnaire used for adults and children.

1a: Adult questionnaire	
**Questions**	**Options to analyse**
**Q1. Are you interested in animal welfare?**	1 (Not at all) - 4 (Very much)
**Q2. In general, do you think zoo animals are happy?**	1 (Not at all) - 4 (Very much)
**Q3. Do you want animals in zoos to be happy?**	1 (Not at all) - 4 (Very much)
**Q4. Which of the following are acceptable in order for zoo animals to be happier? (multiple choice allowed)**	□ Higher entry ticket price □ Fewer animal species to see □ Higher taxes □ Harder to see animals □ Less access to animals □ Gradually closing zoos □ Fewer hours/day the zoo is open
**Q5. What do you think is important for the happiness of animals in zoos?**	Free response
**Q6. Do you agree that it is okay to use the following animals as live prey in zoos?**	**Rabbit** □Agree □Disagree □Depends on the situation □ Don’t know **Mouse** □Agree □Disagree □Depends on the situation □ Don’t know **Chicken** □Agree □Disagree □Depends on the situation □ Don’t know **Frog** □Agree □Disagree □Depends on the situation □ Don’t know **Goldfish** □Agree □Disagree □Depends on the situation □ Don’t know **Sardine** □Agree □Disagree □Depends on the situation □ Don’t know **Octopus** □Agree □Disagree □Depends on the situation □ Don’t know **Crayfish** □Agree □Disagree □Depends on the situation □ Don’t know **Clam** □Agree □Disagree □Depends on the situation □ Don’t know **Cricket** □Agree □Disagree □Depends on the situation □ Don’t know **Earthworm** □Agree □Disagree □Depends on the situation □ Don’t know
**Q7. If your answers were same across species in Q6, please explain why you made those decisions?**	Free response
**Q8. If your answers differed by species in Q6, were your choices impacted by the following factors? (multiple choice allowed)**	□ Whether you like the species or not □ Whether or not you can empathize with the animal species (whether or not you feel sorry for it) □ Differences in the cognitive abilities of the animal species □ The complexity of the animal’s life and behaviour □ Differences in the ability of animal species to feel pain and suffering □ Whether or not it happens often □ Other characteristics of animals species (Please specify: ） □ Nothing specific □ Other（ ）
**Q9. Do you think the following organizations/persons are fulfilling their responsibilities when it comes to ensuring the happiness of captive animals?**	**National (Government)** □ Yes □ No □ Neither □ Do not know □ Never cared □ Prefer not to say **Zoo management** □ Yes □ No □ Neither □ Do not know □ Never cared □ Prefer not to say **Zoo keepers** □ Yes □ No □ Neither □ Do not know □ Never cared □ Prefer not to say
**Age**	If you are under 18, please write the exact age（ ）year old □18 – 29 □ 30 – 39 □ 40 – 49 □ 50 – 59 □ over 60
**Nationality**	□ British □ Other ( )
**Sex**	□ Male □ Female □ Non-binary □ Other
**Final Education**	□ Junior high/High school □ Vocational college □ University or higher □ Other
**Are you interested in animals?**	1 (Not at all) - 4 (Very much)
**What types of connections do you currently have with animals? (multiple choice allowed)**	□ None in particular □ Keep animals at home □ Work with animals □ Visit zoos/aquariums frequently □ Animal-related hobby (e.g., fishing, bird watching)
**Do you actively search for information related to science?**	□ Yes □ No □ Neither
1-b: Children questionnaire	
**Questions**	**Options**
**Q1. What do you think is important for the happiness of animals in zoos?**	Free response
**Q2. Some animals are predators – they eat other live animals in the wild (like a cat eats a mouse, or a bird eats an insect). Do you think that it is ok to feed these live animals to other animals in the zoo?**	**Rabbit** □Agree □Disagree □Depends on the situation □ Don’t know **Mouse** □Agree □Disagree □Depends on the situation □ Don’t know **Chicken** □Agree □Disagree □Depends on the situation □ Don’t know **Frog** □Agree □Disagree □Depends on the situation □ Don’t know **Goldfish** □Agree □Disagree □Depends on the situation □ Don’t know **Sardine** □Agree □Disagree □Depends on the situation □ Don’t know **Octopus** □Agree □Disagree □Depends on the situation □ Don’t know **Crayfish** □Agree □Disagree □Depends on the situation □ Don’t know **Clam** □Agree □Disagree □Depends on the situation □ Don’t know **Cricket** □Agree □Disagree □Depends on the situation □ Don’t know **Earthworm** □Agree □Disagree □Depends on the situation □ Don’t know
**Q3. If your answers were same across species in Q2, why did you think so?**	Free response
**Q4. If your answer differed by species in Q2, why did you think so? (Multiple choice allowed)**	□ Whether you like the species or not □ Whether or not you feel sorry for the animal □ Differences in how clever the animals are □ Because of the animal’s complex life and behaviour □ Differences in the ability of the animal to feel pain and suffer □ Whether or not it happens often □ Other things about the animal (Please specify:) □ Nothing specific □ Other ( )
**Age**	( ) year old
**Nationality (British survey only)**	□ British
**Sex**	□ Male □ Female □ Non-binary □ Other

In the survey, we used the word ‘happiness’ because the concept of ‘animal welfare’ is not prevalent among the Japanese public, and the term conveys positive feelings in both countries. However, we must exercise caution with the term ‘happiness’ as it lacks a specific definition [37] and it remains unclear whether good animal welfare, happiness, and well-being encompass the same meaning [38]. Nevertheless, the concept of animal welfare directly implies the importance of animals’ feelings [e.g., 39,40], and animals that are happy are likely to be experiencing good animal welfare [41]. Moreover, using terms such as ‘welfare’ or ‘well-being’ with a certain definition in the questionnaire might introduce potential bias. Therefore, we opted for the term ‘happiness’ as it is a simple expression directly related to ‘welfare’ or ‘well-being’ in Japan. In many instances, Japanese media or books frequently employ the term ‘happiness’ to elucidate animal welfare for the Japanese public [42].

This study was approved by the ethics committee of the University of Stirling (#7150), Kyoto City Zoo (#2021-KCZ-025 and #2022-KCZ-012), and Japan Monkey Centre (#2021005). The individuals responsible for collecting children’s data in Britain (DAW, MH, and two research assistants) were registered with the Protecting Vulnerable Groups (PVG) scheme, managed by Disclosure Scotland. Anonymous data used for the analyses is openly available at Open Science Framework (https://osf.io/d2gh3/?view_only=77263055c10b4978b1f1e7b2207c8f78).

### Data analyses

Prior to analyses, the data set was cleaned, so that any obvious errors (i.e., where a respondent had answered in a wrong section) were corrected. We used R version 4.3.1 for statistical analyses [43]. The analyses were conducted separately for adults (over 18 years old) and children (6–17 years old). For basic respondent information such as age and gender, we utilized chi-squared tests to examine differences between the two countries. In cases where differences were significant, we conducted residual analyses. We used generalized linear models (GLM) and included age and gender as independent variables to analyse questions 1–3 (Are you interested in animal welfare? In general, do you think zoo animals are happy? Do you want animals in zoos to be happy?) in the adult questionnaire and the number of codes appeared from the free-response (What do you think is important for the happiness of animals in zoos?) in each country. The level of significance was set at p <  0.05 for all analyses. Missing data were replaced with ‘NA’ and analyses were conducted accordingly.

### General attitudes toward zoo-housed animal welfare

To assess differences in Q1–3 (on a 4-point Likert scale, 1: Not at all – 4: Very much), we employed GLM with a Poisson distribution and log-link function. Independent variables included country, age, and gender. To identify the best-fit model, minimizing the Akaike Information Criteria (AIC), we compared AIC values [44]. We examined whether the country factor was included in the final model to discuss differences between the countries. When presenting the data, we displayed the full model rather than the final model [45]. Collinearity was assessed using Variance Inflation Factors (VIF), calculated using the ‘car’ R package [46] and we found no issues in any model (all VIF <  2).

To analyse open questions, we applied thematic analysis methods [47]. The first author (YY) extracted codes through data-driven coding and identified themes. Another author (YI) reviewed the categorizations. Categorizations were adjusted based on the review and subsequent discussions between the two authors. The number of codes per person was then calculated, and we compared it among the groups (British adults/children and Japanese adults/children) using GLM. Country, age, and gender were included as independent variables, with the number of codes per person as the dependent variable. Model selection was based on the comparison of AIC, and collinearity was checked with VIF, confirming no issues.

To analyse the costs (see Q4 in Table 1a, e.g. Higher entry ticket price, fewer animal species to see) that people were willing to accept for improved zoo animal welfare, we used chi-squared tests. In cases where differences were significant, we conducted residual analyses.

### Live prey feeding

To examine differences in responses toward live prey feeding, we calculated the ratio of responses for each species in adults’ and children’s samples in both countries. Wilcoxon’s signed-rank test was used to check differences of the ratio across country-age categories (British adults vs. Japanese adults, British children vs. Japanese children, British adults vs. British children, Japanese adults vs. Japanese children) for each response category (agree/disagree/depends/not sure). The ‘exactRankTests’ R package [48] was employed for these tests.

To analyse the reasons behind respondents’ choices on the acceptability of live prey feedings, we conducted separate analyses for individuals who provided the same response for all species, and those who distinguished amongst them. While there have been numerous discussions on species differences concerning sentience and ethical decisions [49], limited attention has been given to understanding the reasoning behind acceptability of live prey feeding across species. Given that this study represents the first cross-cultural comparison on this issue, we designed an open question to investigate the reasons behind consistent choices. In contrast, a closed question was employed to investigate the reasons for distinguishing amongst species. Our focus was primarily on the analysis of reasons why some species were acceptable to live feed and others were not. To analyse the reasons for consistent responses, the first author (YY) applied thematic analysis methods [47] to extract data-driven codes, and the categorizations were subsequently reviewed by another author (YI). Modifications to the categorizations were made through discussions between the two authors. Due to the small sample size for some country-age groups, no statistical analyses were performed regarding the reasons they disagreed with live prey feedings across all species. Ratios were calculated by dividing the number of each response by the sum of responses.

We conducted chi-squared tests to assess differences in the reasons for inconsistent responses between country-age groups (British adults vs. Japanese adults, British children vs. Japanese children, British adults vs. British children, Japanese adults vs. Japanese children). For significant differences, we performed residual analyses. To analyse the reasons why acceptability to live feed varied by species, we conducted chi-squared tests to check differences between country-age groups. When significant, residual analyses were performed. Ratios were calculated by dividing the number of each response by the sum of responses, and the analyses were conducted.

### Trust toward the governing country, zoos and caregivers

Chi-squared tests were performed to assess differences in trust toward governing country, zoo, and caregivers between countries. For significant differences, residual analyses were conducted.

## Results

### Basic characteristics of respondents

The basic characteristics of respondents, including age, gender, final education, interest in animals, connections with animals, and interest in science, are summarized in S2 Table in S1 File. Although the gender ratio did not differ significantly between the countries (χ^2^ =  2.0, df =  2, p =  0.37), other characteristics, such as age (χ^2^ =  223, df =  4, p <  0.001), final education (χ^2^ =  22, df =  3, p <  0.001), interest in animals (χ^2^ =  219, df =  4, p <  0.001), connections to animals (χ^2^ =  96, df =  4, p <  0.001), and interest in science (χ^2^ =  133, df =  2, p <  0.001), differed significantly in adults. In the children’s sample, the gender ratio showed a slight tendency to have more females in the British than Japanese sample but not significantly (χ^2^ =  5.8, df =  2, p =  0.054). The distribution of age differed significantly between the countries (χ^2^ =  33, df =  11, p <  0.001).

### General attitudes toward zoo animal welfare

**Fig 1:**
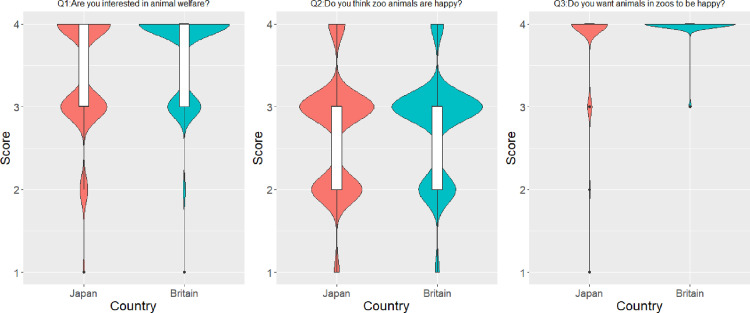
General interests, perceptions, and desires regarding animal welfare between Japanese (red) and British (blue) zoo visitors (Score 1: Not at all – 4: Very much).

Collectively, the British and Japanese samples exhibited comparable responses regarding their interest in and perception of animal happiness, as well as their wishes for the animals to be happy. Country was a factor included in the final model for the question regarding respondents’ interest toward animal welfare. The level of interest toward zoo animal welfare was significantly higher in Britain than in Japan, although the difference was small (Fig 1 and Tables 2 and S3 in S1 File). Country was not included in the final models for the questions regarding respondents’ perceived level of zoo animal welfare and their desire for zoo animals to be happy. In both countries, respondents indicated that they could accept some costs (see Q4 in Table 1a), but the ratio of each response differed across countries (Table 3: χ^2^ =  58, df =  6, p <  0.001). Japanese respondents accepted a higher entrance fee and were more willing to accept less access to animals (fewer opportunities for physical contact) compared to British respondents. On the other hand, British respondents were more accepting of seeing fewer animal species, having more difficulty seeing animals, and having the zoo open for fewer hours per day compared to Japanese respondents.

**Table 2:** Parameter estimates from the models used to explain differences in interest toward animal welfare (Q1), perceived level of animal welfare (Q2), and desire for animal welfare (Q3) between the countries in the adult questionnaire.

Q1	Factor	Estimate	SE	Z	p
	Intercept	1.39	0.50	2.8	0.0056
	Country				
	Britain	0.077	0.033	2.3	0.021
	Age				
	30–39	-0.025	0.045	-0.55	0.58
	40–49	-0.0058	0.046	-0.13	0.90
	50–59	0.0083	0.057	0.15	0.88
	Over60	0.029	0.056	0.52	0.60
	Gender				
	Female	-0.14	0.50	-0.29	0.77
	Male	-0.19	0.50	-0.38	0.71
	Non-binary	-0.029	0.61	-0.048	0.96
Q2	Factor	Estimate	SE	Z	p
	Intercept	1.1	0.58	1.8	0.066
	Country				
	Britain	0.034	0.039	0.89	0.38
	Age				
	30–39	0.024	0.052	0.46	0.65
	40–49	0.033	0.054	0.61	0.54
	50–59	0.018	0.067	0.27	0.79
	Over60	0.070	0.064	1.1	0.27
	Gender				
	Female	-0.097	0.58	-0.17	0.87
	Male	-0.11	0.58	-0.20	0.84
	Non-binary	-0.42	0.76	-0.55	0.58
Q3	Factor	Estimate	SE	Z	p
	Intercept	1.4	0.50	2.8	0.0058
	Country				
	Britain	0.027	0.032	0.85	0.40
	Age				
	30–39	-0.013	0.043	-0.31	0.76
	40–49	0.00068	0.044	0.015	0.99
	50–59	0.0098	0.054	0.18	0.86
	Over60	0.012	0.053	0.22	0.82
	Gender				
	Female	-0.024	0.50	-0.047	0.96
	Male	-0.049	0.50	-0.097	0.92
	Non-binary	-0.0065	0.61	-0.011	0.99

**Table 3:** The results of costs that visitors would find acceptable in each country.

	Fee	Species	Tax	Observation	Less access	Closing	Availability	Total
Japan	555	158	127	143	338	142	352	861
Britain	264	155	70	139	153	78	248	508
	p < 0.001	p < 0.001	N.S.	p < 0.001	p < 0.001	N.S.	p = 0.051	

The figures represent a combination of a boxplot and a violin plot, with a density curve illustrating the data distribution.

Thirty-eight codes emerged from the free responses of factors constituting animal happiness, and are summarized in S4 Table in S1 File. The final model included the factor ‘country’. Generally, British respondents generated more topics than their Japanese counterparts. Mean number of codes per respondent was 2.9 for British adults, 2.3 for British children, 0.71 for Japanese adults, and 1.4 for Japanese children (Tables 4 and S3 in S1 File).

**Table 4:** Parameter estimates from the models used to explain differences in the number of codes extracted from free responses in each country.

Factor	Estimate	SE	Z	p
Intercept	<0.001	1.0	0	1.0
Country				
Britain	1.2	0.044	28	<0.001
Age				
Child	0.14	0.053	2.6	0.0081
Gender				
Female	-0.16	1.0	-0.16	0.87
Male	-0.37	1.0	-0.37	0.71
Non-binary	-0.41	1.1	-0.39	0.70

There was an overlap between the countries, as individuals in both regions frequently wrote about basic codes such as *food*, *natural concept* (e.g., conditions similar to the wild), *sociality*, and *size* (Table 5). However, British respondents focused more on the concepts of increasing stimulation and positive mental aspects than Japanese respondents, and codes related to animal welfare only appeared in Britain. Conversely, Japanese respondents wrote more about *animal life* (e.g., longevity and living) and *stress* than their British counterparts. Although rare, the code of *human welfare* only appeared in Japan. The age at the first appearance of each code is summarized in Table 5, and differences emerged early in childhood.

**Table 5:** The occurrence rate of each code in each country (divided by the number of respondents who answered the free response) and the age at which each code first appeared.

	Rate of each code				Age at first appearance (yr)	
	Japan		Britain		Japan	Britain
Code	Adults	Children	Adults	Children		
Natural concept	0.205	0.074	0.391	0.107	8	11
Other concept	0.158	0.089	0.132	0.175	6	7
Philosophical	0.019	0.007	0.007	0.000	7	18-29
Increase stimulation	0.000	0.007	0.113	0.078	17	10
Decrease stimulation	0.028	0.007	0.093	0.019	9	16
Choice	0.009	0.000	0.033	0.000	18–29	18–29
Freedom	0.101	0.022	0.029	0.019	13	7
Individual traits	0.012	0.000	0.015	0.029	18–29	7
Human mind and attitude	0.087	0.126	0.121	0.214	7	16
Animal life	0.012	0.015	0.007	0.000	7	18–29
Physical health	0.017	0.022	0.011	0.019	11	10
Mental health	0.014	0.007	0.091	0.058	6	7
Positive mental	0.009	0.000	0.049	0.039	30–39	7
Negative mental	0.002	0.000	0.015	0.000	18–29	18–29
Stress	0.120	0.096	0.015	0.019	8	16
Behaviours	0.007	0.015	0.077	0.010	13	15
Animal welfare	0.000	0.000	0.046	0.010	NA	17
Size	0.184	0.089	0.547	0.320	6	6
Food	0.099	0.370	0.305	0.573	6	6
Safety	0.024	0.015	0.190	0.155	11	6
Play	0.012	0.089	0.022	0.097	6	7
Sleep	0.007	0.015	0.004	0.029	7	6
Sociality	0.042	0.052	0.152	0.175	6	7
Hygiene	0.024	0.030	0.035	0.049	6	10
Temperature	0.007	0.007	0.026	0.029	12	9
Veterinary	0.005	0.000	0.062	0.019	30–39	7
Resources (finance, staff number and skill)	0.038	0.000	0.029	0.000	18–29	18–29
Scientific evaluations	0.000	0.000	0.020	0.000	NA	18–29
Breeding	0.005	0.000	0.022	0.010	30–39	17
Environmental improvement	0.005	0.000	0.137	0.010	30–39	12
Understanding animals	0.005	0.000	0.009	0.000	40–49	18–29
Human animal relationships	0.026	0.015	0.068	0.019	7	16
Visitors	0.050	0.007	0.108	0.049	15	16
Human welfare	0.005	0.000	0.000	0.000	30–39	NA
Exercise	0.005	0.000	0.007	0.019	40–49	7
Animal abuse	0.002	0.022	0.002	0.010	7	6
Exhibition	0.007	0.037	0.013	0.000	6	18–29
Other	0.026	0.015	0.064	0.019	8	12

The thickness of the colours corresponds to the rates of each code. Thicker colour represents higher rates.

### Live prey feeding

**Fig 2:**
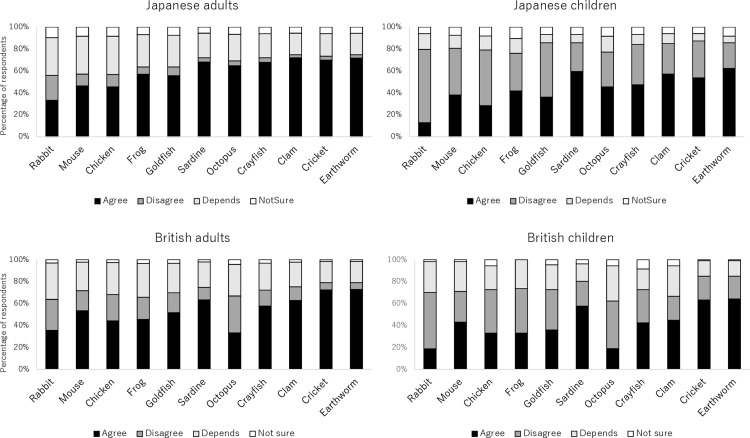
Percentage of respondents choosing *agree*, *disagree*, *depends*, and *not sure* for live prey feeding of each species across Japanese and British adults and children (6–17 years).

The responses toward live prey feeding were similar between countries, except for those toward octopus (Fig 2). The percentage of British respondents who agreed that live prey feeding of octopus was acceptable was low, even lower than for rabbits. Despite this clear difference in octopus responses, the rate of agreement did not differ significantly between the countries for adults (V =  14, p =  0.10) or children (V =  26, p =  0.58). However, differences emerged between adults and children in both countries, with children showing generally lower acceptability than adults (Britain: V =  66, p <  0.001; Japan: V =  66, p <  0.001). The percentages of *disagree*, *depends* and *not sure* were different between the countries and adults/children. Japanese respondents provided more ambiguous responses (*depends*/*not sure*) than British respondents did. The percentage of people who disagreed with live prey feeding was higher in British respondents than in Japanese respondents (adults: V =  66, p <  0.001; children: V =  9, p =  0.032) and in children than in adults (Britain: V =  0, p <  0.001; Japan: V =  0, p <  0.001). The percentage of child respondents who chose *depends* was higher in Japan than in Britain (adults: V =  27, p =  0.64; children: V =  66, p <  0.001) and not different between adults and children in Britain but lower in children than in adults in Japan (Britain: V =  52, p =  0.098; Japan: V =  66, p <  0.001). The percentage of respondents who chose *not sure* was higher in Japan than in Britain (adults: V =  0, p <  0.001; children: V =  2, p =  0.0029) and not different between adults and children (Britain: V =  19, p =  0.23; Japan: V =  21, p =  0.32). There was a similar percentage of respondents making consistent answers (i.e., agree across all species) both in Britain and Japan. Thirty-one percents of Japanese adults, 24% of British adults, 9.7% of Japanese children, and 6.5% of British children agreed on the acceptability of live prey feeding of all species. The percentages of those consistently disagreeing on the acceptability of live prey feeding of all species was much lower (5.3% of British adults, 2.1% of Japanese adults, 14% of British children and 14% of Japanese children).

**Fig 3:**
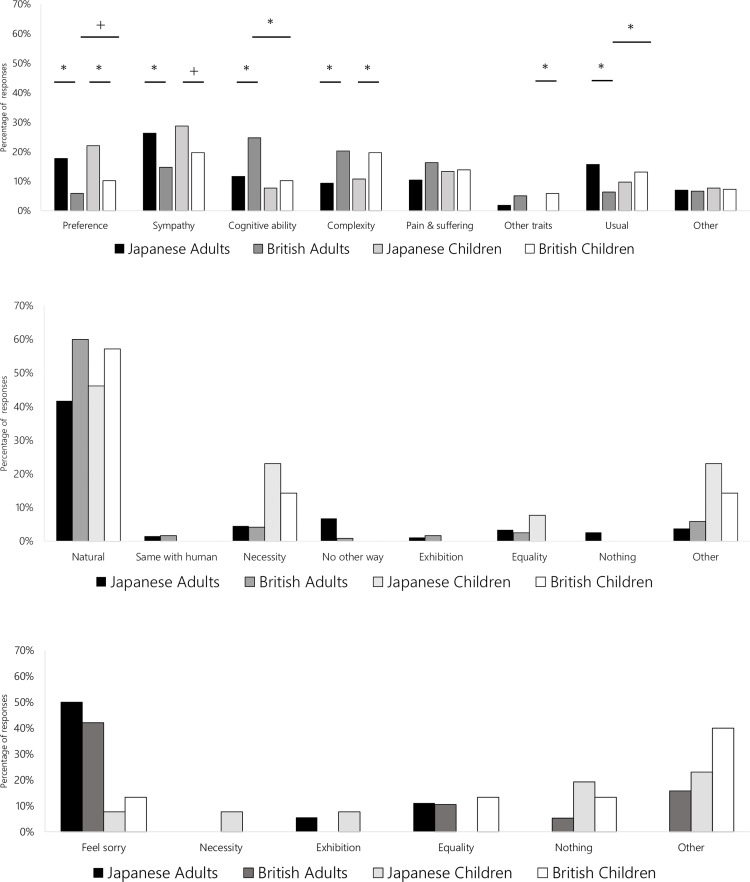
(a) Reasons for why British and Japanese respondents consistently agreed on the acceptability of live prey feeding (Number of respondents: Japanese adults: N =  266, British adults: N =  120, Japanese children: N =  13, British children: N =  7) *  p <  0.05, +  0.05 <  p <  0.1. (b) Reasons for why British and Japanese respondents consistently disagreed on the acceptability of live prey feeding (Number of respondents: Japanese adults: N =  18, British adults: N =  26, Japanese children: N =  19, British children: N =  15). (c) Reasons for why British and Japanese respondents made inconsistent responses on the acceptability of live prey feeding (Number of respondents: Japanese adults: N =  429, British adults: N =  269, Japanese children: N =  96, British children: N =  76).

The reasons why respondents consistently agreed to the acceptability of live prey feeding (regardless of species) were similar between British and Japanese adults (Fig 3a). The most common (open question) answer code was *natural* for adults and children in both countries. The most common answer for the reasons why respondents consistently disagreed to the acceptability of live prey feeding was *feel sorry* for Japanese adults and children, with no clear trend for British individuals (Fig 3b).

The reasons for inconsistent choices for the acceptability of live feeding (i.e., distinguished between acceptability across species) differed between countries (Fig 3c: Adults: χ^2^ =  157, df =  7, p <  0.001; Children: χ^2^ =  26, df =  7, p <  0.001) and adults/children in Britain (χ^2^ =  23, df =  7, p =  0.0016) but not in Japan (χ^2^ =  13, df =  7, p =  0.074). Japanese respondents tended to choose options reflecting human perspectives more often than British respondents (*Preference*: Adults: p <  0.001, Children: p =  0.0049; *Sympathy*: Adults: p <  0.001, Children: p =  0.062). Conversely, British respondents tended to choose options reflecting animal perspectives more than Japanese respondents did in adults (*Cognitive abilities*: p <  0.001, *Complexity*: p =  0.0043, *Pain and suffering*: p =  0.0019, *Other traits*: p =  0.0017) and in children (*Cognitive abilities*: p =  0.42, *Complexity*: p =  0.023, *Pain and suffering*: p =  0.89, *Other traits*: p <  0.001). British adults tended to choose *cognitive abilities* more than British children did (p <  0.001), while British children tended to choose *usual* more than British adults did (p =  0.0063). Japanese adults tended to choose *usual* more than British respondents did (p <  0.001).

### Trust toward governing countries, zoos and caregivers

**Fig 4:**
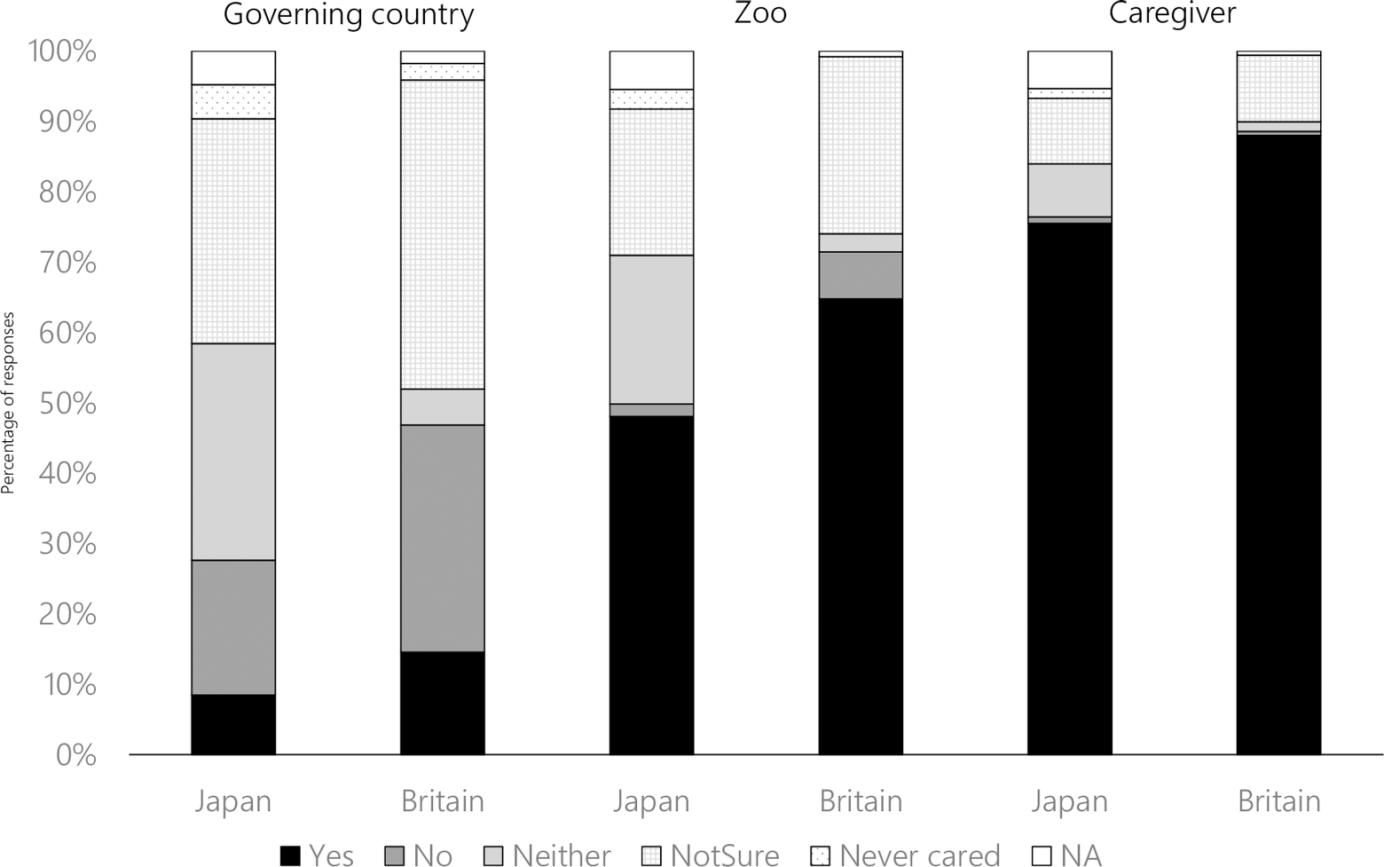
Trust by Japanese and British zoo visitors toward governing countries (Japan and Britain), zoos and caregivers (Number of respondents: Japanese adults: N =  861, British adults: **N** =  508).

Fig 4 shows the percentage of respondents who chose each response. In terms of trust toward the governing countries, the ratio of respondents who chose each response differed significantly between the countries (Governing countries: χ^2^ =  158, df =  5, p <  0.001; Zoo: χ^2^ =  151, df =  5, p <  0.001; Caregivers: χ^2^ =  57, df =  5, p <  0.001). The percentage of respondents who chose *Yes*/ *No*/ *Not sure* was higher in Britain than in Japan (Yes: p <  0.001; No: p <  0.001; Not sure: p <  0.001). Japanese respondents tended to choose the options of *Neither*/ *Never cared* more than British respondents did (Neither: p <  0.001; Never cared: p =  0.021). The trends were similar for trust toward the zoo (Yes: p <  0.001, No: p <  0.001, Neither: p <  0.001, Not sure: p =  0.059, Never cared: p <  0.001). The percentage of respondents who chose *Yes* for the trust toward caregivers was higher in Britain than in Japan (p <  0.001). Japanese respondents chose *Neither* and *Never* cared more than British respondents did (Neither: p <  0.001, Never cared: p =  0.0075). There was no difference between the countries in the percentage of respondents who chose *No* (p =  0.50) and *Not sure* (p =  0.92). The percentage of respondents who chose *Yes* gradually increased from nation, zoo, to caregivers in both countries.

## Discussion

This study compared the attitudes of British and Japanese zoo visitors towards zoo animal welfare and related issues. We found that visitors from both countries have a high interest in animal happiness. Despite the fact that the two countries differ in terms of animal welfare policy and legislation (e.g., the World Animal Protection [36] concluded that animal welfare policies were significantly better in the UK compared to Japan from the perspective of animal welfare), general public interest toward animal happiness is high in both Britain and Japan, and any differences in interest were very small. Both sets of visitors were willing to accept some costs (e.g., financial or limited access to viewing in zoos) for the sake of the well-being of the animals, although exactly what they were willing to accept differed between the two countries. Whether their evaluations match with the actual animal welfare states in each country should be investigated in the future.

In relation to what is important for animal welfare, *natural concept*, specific environmental features (such as *enclosure size* and *food*), and *human mind and attitudes* were frequently mentioned in both countries. This aligns with previous studies suggesting that subjective experience of animals may not provide a comprehensive understanding when people assess concepts related to animal welfare, happiness, or well-being, with naturalness being identified as a crucial factor in their judgement [4,19,50–52]. People’s assessments consist of a combination of animal conditions and methods aimed at enhancing those conditions. These shared codes can be considered ‘intuitive’, and exhibiting minimal cultural influence. While animal mental states were commonly assumed to be important features in assessing their welfare among scientists [39,40,53], the priority given to animal mental states and how they are described differed across countries in our study. Similar to a previous study on farm animal welfare where humane treatment was identified as an important feature [51], *human mind and attitudes*, as well as *human-animal relationships*, were mentioned in Britain and Japan. Visitor-related issues, such as *space away from visitors* and *manners of visitors*, were also frequently mentioned in both countries. However, opposing views were sometimes observed within an identified code, as some respondents expressed the importance of keeping a distance from animals with statements like ‘*Wild animals should have as little human contact as possible*’ and ‘*Keeping humans at a distance*,’ while others advocated for the opposite, stating, ‘*Taking care of them carefully and in close proximity*’. It is unclear whether observed differences are attributable to cultural, individual, or contextual factors.

Distinct differences between Britain and Japan were evident in certain extracted codes. Specifically, only British respondents mentioned terms such as *animal welfare* and *scientific evaluations*. They used terms such as *increase stimulation*, *positive mental* and *environmental improvement* more frequently than Japanese respondents. In contrast, Japanese respondents mentioned terms, such as *stress*, *freedom*, and *life*, more often compared to British respondents. Although a small proportion did so, only Japanese participants mentioned *human welfare*, referring to the treatment of humans who work with or observe animals. In general, the number of codes mentioned per person was higher in Britain than in Japan. British respondents mentioned more concrete factors compared to their Japanese counterparts. These differences may align with the general distinctions between animal welfare, which focuses on the physical and mental states of animals based on objective criteria, and animal aigo, which centers on human sentiment and protecting animal life [12]. We anticipated that responses from children would not differ significantly between countries due to potentially limited cultural influence. While this was true for some basic issues common to children in both countries, differences emerged in early childhood, with variations in the timing of when specific codes first appeared in each country. The most common codes remained consistently prevalent in both countries even after individuals reached adulthood. For instance, the code *life* appeared at the age of 7 in Japan and 18–29 years in Britain. Similarly, the code *positive mental* appeared at the age of 7 in Britain and 30–39 years in Japan. These findings suggest that these divergent ideas may take shape over an extended period, potentially influenced by learning at school or home [22]. Zoos and aquariums can be important places for such education, especially considering that children frequently visit them.

The results of the live prey feeding survey revealed both similarities and differences between Britain and Japan. General trends were consistent, except for the treatment of octopus. Differences between adults and children were also comparable across countries, with children generally responding more negatively to live prey feeding. The observation that younger respondents exhibit more empathetic responses is corroborated by previous studies [22]. The present study was conducted shortly after the passage of the Animal Welfare Act 2022 in the UK, which includes functions related to the impact of government policy on the welfare of animals as sentient beings [54]. All vertebrates and some invertebrates (cephalopod molluscs such as octopus and squid and decapod crustaceans such as crabs and lobsters) were classified as sentient beings in the new legislation. Live prey feeding of vertebrates is discouraged under the UK Animal Welfare Act 2006 [55] and Zoo Licensing Act [27], but now extends to these invertebrates. However, in our study, British responses did not differ significantly from Japanese responses, regarding crayfish (a decapod crustacean) but did differ regarding octopuses (a decapod mollusc). There were certain percentages of respondents who consistently agreed to live prey feeding across all species, and the most common rationale given was that it is ‘natural’ for both British and Japanese respondents. The fact that 35% of British adults agreed to live prey feeding of rabbits was consistent with a previous study conducted 27 years ago [30], which reported that 32% of respondents agreed to live prey feeding of rabbits to cheetahs when they were on exhibit. Even though we did not specify whether the live feeding would take place on or off exhibit, people’s attitudes do not appear to have changed significantly. The discrepancies between human attitudes and the legislation in their country were consistent with a previous study on the acceptability of live prey of aquatic species in the UK and US [17].

Despite significant overlap in responses regarding the acceptability of live feeding of various species in both countries, it is interesting to note that the reasons for discrimination between species differed between the countries. Japanese respondents were more likely to base their choices on how they felt about the animals (e.g., personal preference and sympathy) than British respondents. In contrast, British respondents were more likely to base their choices on what they know about animals (e.g., cognitive abilities and complexities of life and behaviours) compared with Japanese respondents. This trend was consistent in both adult and child samples, reflecting the attitudes represented in the laws of each country; species distinction based on animal characteristics in Britain (e.g., sentience) and on human sentiment (i.e., feelings towards animals) in Japan. As noted above, one notable difference between the countries was the treatment of octopuses. Recent English language documentaries (such as The Octopus in my House [56]; My Octopus Teacher [57]) depict octopuses as highly intelligent. This may be one reason why British respondents prioritize octopus for protection, considering that ethical decision-making about animals is based on the likelihood of sentience, and the cognitive abilities and complexities of life and behaviours of animals in Britain [58,59]. In contrast, the link between cognitive abilities and ethical treatment is not explicitly discussed even in Japanese animal welfare textbooks and laws [9,60]. Octopuses are considered as a common food by most Japanese people. This point is crucial as it indicates that different cultural approaches are necessary to change attitudes. In this example, if British respondents learn about the intelligence of the animal, they may change their attitudes towards their welfare and protection. However, it may not be as effective in Japan, given the differences in the underlying reasons for making ethical decisions. It would be interesting to investigate the effectiveness of presenting information about animals in culturally specific ways to test its impact on changing public attitudes in each country.

In terms of trust in governing countries, zoos and caregivers, trends were similar between the two countries. However, the fact that Japanese respondents were more likely to choose ambiguous options (e.g., *not sure*) made it challenging to draw concrete conclusions. Nevertheless, some interesting trends were identified. Firstly, very few British respondents believe that their government fulfils its responsibilities over animal happiness, despite the existence of extensive laws and regulations in the UK such as the Animal Welfare Act and Zoo Licensing Act [27,54,55,61]. Considering the differences in the development of animal welfare laws in the two countries, the observed differences in trust were surprisingly small. While international organizations utilize legislative systems to rank animal welfare in each nation [36], the general public’s evaluations are not likely based on a thorough understanding of the legislation but on cultural attitudes towards animal welfare and general trust in the government. Rank et al. (2018) suggested that ethical integrity (e.g., how much animal needs are met) is important for organizational trust in zoos and aquariums [33]. Trust in zoos and caregivers did not differ significantly between Britain and Japan, suggesting that the zoos in our study manage to earn trust from visitors. However, since these surveys were conducted among zoo visitors, the trend may differ if the survey were conducted outside the zoos. The study conducted at Edinburgh Zoo found that zoo visitors had a more positive perception of zoo animals than the general public outside of the zoo environment [62].

This study has several limitations. Firstly, the sample size and age distribution between the British and Japanese respondents differed. The age distribution at Kyoto City Zoo was similar to previous surveys at the zoo [63,64], so likely representative of visitors to Kyoto City Zoo. Secondly, as this was an onsite questionnaire conducted in a limited number of zoos, our study does not represent entire countries or include the opinions of people who have no interest in zoos. Even within the same country, attitudes and laws regarding animal welfare can vary [65]. Reade and Waran (1996) [62] noted differences in the perception of animals when surveys were conducted on the streets compared to at the zoo. Future studies should aim to collect data from a more diverse population. Thirdly, this study was explorative in nature and covered various topics such as human perception, legislation, and trust. Although we were interested in the interrelationships among these, we could not clearly distinguish between their relative contributions to overall animal welfare attitudes. Despite these limitations, we believe that this study is an important step in facilitating future cross-cultural studies. Understanding cultural attitudes and their origins is crucial for understanding and discussing animal welfare issues in their appropriate societal context.

This study demonstrated a significant overlap in societal perceptions of animal welfare between two culturally distinct nations. There was a shared interest in and perception of animal welfare and trust in British and Japanese respondents suggesting that there may be some universal trends in animal welfare perceptions. Specifically, these basic trends indicate that individuals possess a fundamental motivation to care for non-human animals, with the concept of ‘naturalness’ playing a pivotal role in shaping both general attitudes and practices such as live prey feeding. However, some differences between British and Japanese respondents were evident. British respondents more often mentioned animal focused objective traits, while Japanese people frequently highlighted human focused perspectives and feelings, both in their general attitudes toward animal happiness and live prey feeding. Japanese people have a tendency to prioritize mood or sentiment in their attitudes toward animals, as reflected in the culture of ‘Animal Aigo.’[12]. This is not limited to animal issues but is also highlighted in the context of basic ethical attitudes toward humans [66] and science [67]. It is crucial to be aware of these ‘small’ differences when discussing animal welfare issues, as even using the same term ‘animal welfare’ may evoke different associations, complicating the situation. Although understanding public opinion is important, it does not always directly align with the scientific evidence underpinning animal welfare. Therefore, efforts should be made to identify and bridge the gaps between public attitudes and knowledge and the current scientific evidence. Finally, educating children may be important as our study also revealed that some differences emerge in early childhood. Further studies are needed to explore specific educational methodologies and whether attitudes can change with the provision of animal welfare information.

## Supporting information

S1 File S1 Table. Questionnaires for all the participants. S2 Table. Basic characteristics of respondents. S3 Table. AIC results of GLM analyses for Q1-3 and the Number of Code appeared from the Q5. S4 Table. Explanations of codes obtained from thematic analyses of free responses (What do you think is important for the happiness of animals in zoos?).
(XLSX)
